# Tooth Erosion and Eating Disorders: A Systematic Review and Meta-Analysis

**DOI:** 10.1371/journal.pone.0111123

**Published:** 2014-11-07

**Authors:** Ana Paula Hermont, Patrícia A. D. Oliveira, Carolina C. Martins, Saul M. Paiva, Isabela A. Pordeus, Sheyla M. Auad

**Affiliations:** Department of Pediatric Dentistry and Orthodontics, Faculty of Dentistry, Universidade Federal de Minas Gerais, Belo Horizonte, Minas Gerais, Brazil; University of North Carolina at Chapel Hill, United States of America

## Abstract

**Background:**

Eating disorders are associated with the highest rates of morbidity and mortality of any mental disorders among adolescents. The failure to recognize their early signs can compromise a patient's recovery and long-term prognosis. Tooth erosion has been reported as an oral manifestation that might help in the early detection of eating disorders.

**Objectives:**

The aim of this systematic review and meta-analysis was to search for scientific evidence regarding the following clinical question: Do eating disorders increase the risk of tooth erosion?

**Methods:**

An electronic search addressing eating disorders and tooth erosion was conducted in eight databases. Two independent reviewers selected studies, abstracted information and assessed its quality. Data were abstracted for meta-analysis comparing tooth erosion in control patients (without eating disorders) vs. patients with eating disorders; and patients with eating disorder risk behavior vs. patients without such risk behavior. Combined odds ratios (ORs) and a 95% confidence interval (CI) were obtained.

**Results:**

Twenty-three papers were included in the qualitative synthesis and assessed by a modified version of the Newcastle-Ottawa Scale. Fourteen papers were included in the meta-analysis. Patients with eating disorders had more risk of tooth erosion (OR = 12.4, 95%CI = 4.1–37.5). Patients with eating disorders who self-induced vomiting had more risk of tooth erosion than those patients who did not self-induce vomiting (OR = 19.6, 95%CI = 5.6–68.8). Patients with risk behavior of eating disorder had more risk of tooth erosion than patients without such risk behavior (Summary OR = 11.6, 95%CI = 3.2–41.7).

**Conclusion:**

The scientific evidence suggests a causal relationship between tooth erosion and eating disorders and purging practices. Nevertheless, there is a lack of scientific evidence to fulfill the basic criteria of causation between the risk behavior for eating disorders and tooth erosion.

## Introduction

The incidence of eating disorders (EDs) has increased over the past decade, both in males and females [1]. These conditions are associated with significant functional impairment and serious physical and psychological consequences due to an excessive preoccupation with body weight or shape. The mortality and morbidity rates associated with EDs are among the highest of any mental disorders [2].

Medical complications from EDs may affect any organ and be life-threatening [3]. Tooth erosion (TE) has been considered an oral manifestation of EDs associated with vomiting practices [3]–[6]. TE is a complex and multifactorial condition characterized by a progressive and irreversible loss of tooth structure due to a chemical process without bacterial involvement. It is clinically detectable as thinner enamel with chamfered ridges, cupped cusp tips and grooved incisal edges, sometimes with dentine exposure [7], [8].

Dentists usually monitor their patients on a regular basis, sometimes throughout their childhood and adolescence. Therefore, they may be the first health professionals to suspect EDs, due to their oral implications, contributing to the patient's early referral to specific treatment [3], [9]. Nevertheless, even with the increasing prevalence of EDs, the causal effect between these EDs and TE has not been thoroughly discussed in literature.

Hill's criteria of causation [10] must be considered in contemporary epidemiology and consist of nine items: strength of association, consistency, specificity, temporality, dose response, experimental evidence, biological plausibility, coherence, and analogy [11].

An evaluation of Hill's criteria of causation applied to the possible causal relationship between EDs and TE suggests specificity between both conditions (patients who suffer from EDs may present TE), temporality (the cause – EDs – occur before the consequence – TE), biological plausibility (vomiting practices related to the EDs causes an acid attack to tooth enamel), coherence (one cause is specific to one effect), and analogy (other diseases or exposures, such as acidic food or gastroesophageal reflux can cause TE).

Nevertheless, all the points raised before have to be systematically discussed and analyzed before drawing inferential causal conclusions. Furthermore, it is important to evaluate the strength of evidence of such an association and of the dose-response relationship, and search for experimental evidence. The aim of the present systematic review and meta-analysis was to search for scientific evidence of the following clinical question: Do EDs increase the risk of TE?

## Materials and Methods

### Search Strategy

The inclusion criteria for this systematic review were: epidemiological studies (cross-sectional, case-control, cohort and clinical trials) concerning etiological factors and/or the prevalence of TE and its association to any type of EDs (bulimia, anorexia, binge-eating, dysmorphic body disorder, vomiting, hyperphagia) in humans (Checklist S1 presents the PRISMA checklist for systematic reviews).

The exclusion criteria were: unrelated epidemiological studies (other outcome rather than TE), reviews, studies reporting vomiting habits not related to eating disorders, case reports/case series/letters to the editor, laboratorial studies (in vitro studies, extracted teeth, fossils), studies reporting dental treatment, dental materials, knowledge concerning TE, epidemiological studies that did not associate EDs with TE, studies with self-report of TE and infeasibility of extracting data.

The search was conducted in May 2011 and updated in June 2014 by three reviewers (APH, PADO and CCM) in eight different databases: MEDLINE through Pubmed (http://www/pubmed.gov), Web of Science (http://www.isiknowledge.com), Cochrane Library (http://www.cochrane.org/index.htm), Clinical Trials (http://www.clinicaltrials.gov), Current Controlled Trials (http://www.controlled-trials.com), The National Institute for Health and Clinical Excellence (http://www.nice.org.uk), and Lilacs and the Brazilian Library of Dentistry (BBO) through Virtual Health Library (Bireme, Latin America) (www.bireme.br). No restrictions were placed on language or year of publication.

The following search strategy was used in the MEDLINE, Web of Science and Cochrane: ((non-carious cervical lesions OR non-carious cervical lesions OR non-carious cervical lesions OR tooth wear [Mesh] OR dental wear OR tooth erosion [Mesh] OR tooth erosion* OR dental erosion OR dental enamel [Mesh] OR dental enamel OR enamel erosion) AND (anorexia [Mesh] OR anorexia OR anorexia nervosa [Mesh] OR anorexia nervosa OR bulimia [Mesh] OR bulimia OR bulimic eating disorder* OR bulimia nervosa [Mesh] OR eating disorders [Mesh] OR eating disorder* OR binge-eating disorder [Mesh] OR body dysmorphic disorders [Mesh] OR hyperphagia [Mesh] OR binge-eating/vomiting OR vomiting [Mesh] OR vomiting OR risk factors [Mesh] OR pathology [Mesh] OR eating habits) NOT (“animals”[Mesh] NOT “humans”[Mesh])). In MEDLINE, the search was limited to “humans”.

In Lilacs, BBO, Clinical Trials, Current Controlled Trials and The National Institute for Health and Clinical Excellence the search was conducted using combined keywords: “tooth erosion”, “dental erosion”, “enamel erosion”, “anorexia”, “bulimia”, “eating disorder”.

**Figure 1:**
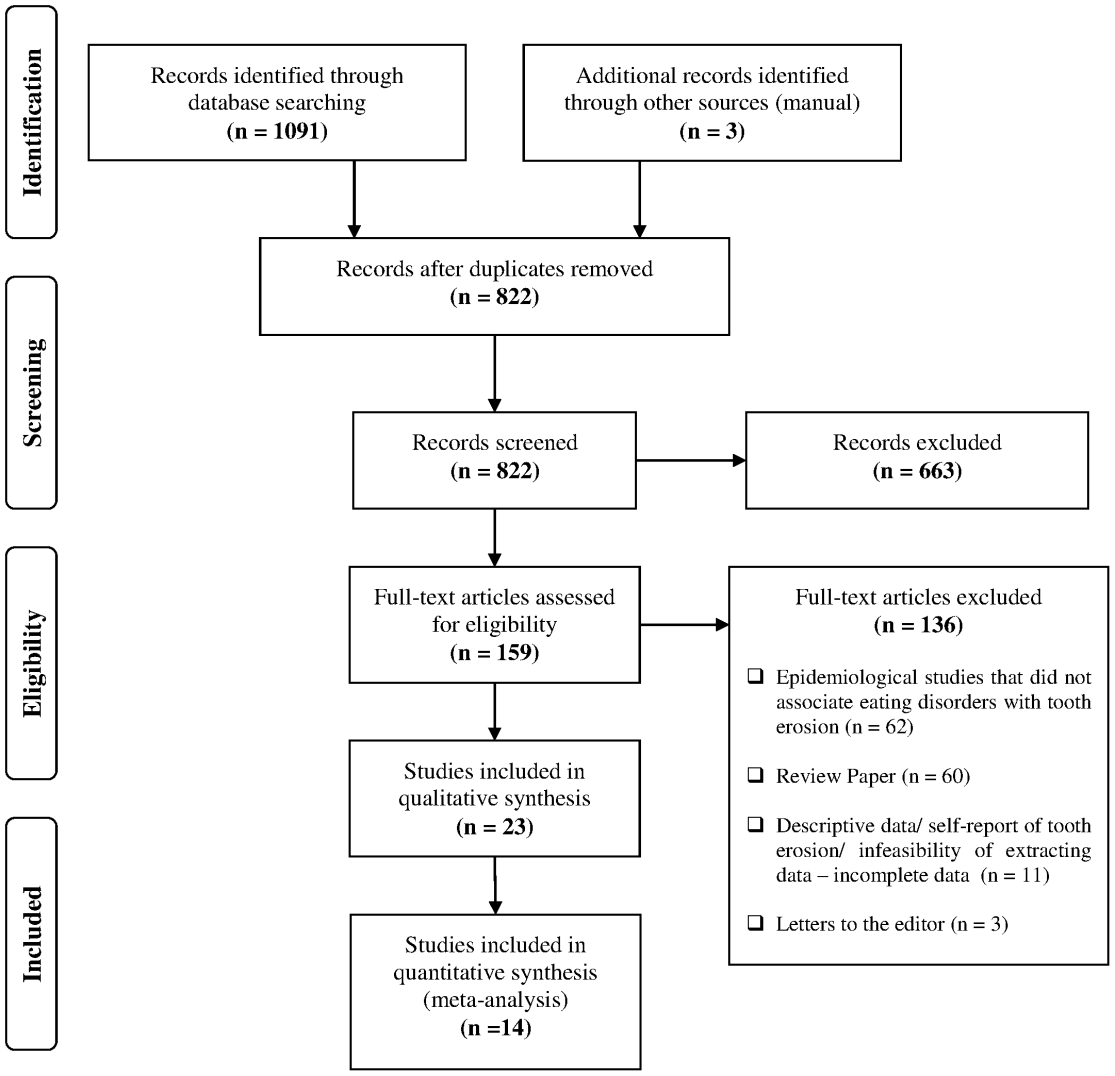
Screening of articles. Four-phase PRISMA flow-diagram for study collection [38], showing the number of studies identified, screened, eligible, and included in the review and meta-analysis.

The electronic search retrieved 1094 abstracts and titles (Figure 1). Reference Manager Software (Reference Manager, Thomson Reuters, version 12.0.3) was used to organize the studies. After the duplicate references were removed, a total of 822 abstracts and titles were read and analyzed by two independent and calibrated reviewers (APH and PADO). As a calibration exercise, the reviewers thoroughly discussed the criteria and applied them to a sample of 20% of the retrieved studies to determine inter-examiner agreement. After adequate agreement was achieved (Kappa: 0.72 to 0.77) all the studies were read by the reviewers independently. Disagreements were resolved by consensus and by the supervision of the gold standard (CCM). If relevant data was missing or if the paper was not available, the primary authors were contacted for additional information/article request.

Systematic reviews, theoretical reviews and additional articles of potential relevance were also manually searched. Grey literature was searched from BBO, which retrieved thesis and monographies, and from MEDLINE, which retrieved abstracts recently presented in congress. During the abstracts' analysis all studies addressing risk factors for TE were selected, even when EDs were not mentioned, in order to search for relevant data not reported in the abstract. The exclusion criteria for abstract and title selection are detailed in Figure 1. Among the 822 records screened, 159 were selected for full text analysis (Table S1 presents excluded studies from full text analysis).

### Data Extraction

Data extraction was conducted by two independent reviewers (APH and PADO) and supervised by the gold standard (CCM). The data analysis is described in Tables 1 and 2. The main analyzed outcome was that TE and EDs were extracted as categorical variables based on authors' descriptions. Extraction was based on non-exposure to risk factor vs. exposure, as follows:

**Table 1:** Quality assessment criteria used for cross-sectional studies through a modified version of Newcastle-Ottawa Scale for case-control studies.

		CASE-CONTROL STUDIES AND THEIR ASSESSMENT RATINGS											
	Dynesen et al., 2008 [21]	Ohrn et al., 1999 [35]	Järvinen et al., 1991 [28]	Johansson et al., 2012 [4]	Emodi-Perlman et al., 2008 [22]	Rytömaa et al., 1998 [20]	Robb et al., 1995 [19]	Milosevic & Slade, 1989 [32]	Jones & Cleaton- Jones, 1989 [31]	Howat et al., 1990 [18]	Sivolella et al., 2000 [33]	Greenwood et al., 1988 [30]	Touyz et al., 1993 [34]
**SAMPLE SELECTION CRITERIA**													
**1) Diagnosis of eating disorders** a) Clinical examination or medical record with validated instrument, or referred from a hospital ★ b)Without clinical examination, or based on self-reports c) No description	★	★	★	★	★	★	★	★	★	★	★	★	★
**2) Representativeness and selection of the patients suffering from eating disorders (cases)** a) Patients with eating disorders selected from a defined catchment area, in a defined hospital or clinic, health maintenance organization, communities or random sample, sample calculation ★ b) Potential for selection biases or not satisfying requirements in part (a) c) No description	★	★	★	★	★	★	★	★	★	★	★	★	c
**3) Selection of participants without eating disorders (controls)** a) Participants selected from a defined catchment area, communities or random sample, sample calculation ★ b) Not satisfying requirements in part (a) c) No description	★	★	★	★	★	★	★	★	★	★	c	★	c
**4) Definition of controls** a) no history of disease (eating disorder) ★ b) no description of source/ not stated if the patient was healthy/ self-report of eating disorders	★	★	★	★	★	★	★	b	★	★	★	b	b
**COMPARABILITY OF CASES/ CONTROLS ON THE BASIS OF THE DESIGN OR ANALYSIS**													
**1) Control for confounders** a) The exposure of interest (tooth erosion) is adjusted for the one confounder ★ b) The exposure of interest (tooth erosion) is adjusted for two or more confounders ★★ c) No description related to the adjustment analysis for confounding factors	★★	★★	★★	★	c	c	c	c	c	c	c	c	c
**EVALUATION OF TOOTH EROSION**													
**1) Diagnosis of tooth erosion** a) Clinical examination reporting the use of a tooth erosion index/ report of observer agreement – kappa ★ b) Satisfied requirements in part (a) and the examiner was blinded to case/ control status ★★ c) Based on self-reports or not satisfying requirements in part (a/b) d) No description	★	★	★	★	★★	★★	★	★★	★	c	c	c	c
**2) Same method of evaluation for cases and controls** a) Yes ★ b) No	★	★	★	★	★	★	★	★	★	★	★	★	★
**3) Response rate** a) Rate of sample loss ≤20% ★ b) Rate of sample loss >20% c) Not stated	★	★	★	★	★	★	★	★	★	★	★	★	★
**SUMMARY SCORE (Stars)**	9/10 (high)	9/10 (high)	9/10 (high)	8/10 (high)	8/10 (high)	8/10 (high)	7/10 (high)	7/10 (high)	8/10 (high)	6/10 (high)	5/10 (low)	5/10 (low)	3/10 (low)

Not all studies described all variables. Confounders were extracted and described as if they were evaluated in multivariate analysis.

**Table 2:** Quality assessment criteria used for cross-sectional studies through a modified version of Newcastle-Ottawa Scale for observational studies.

	CROSS-SECTIONAL STUDIES AND THEIR ASSESSMENT RATINGS									
	Hermont et al., 2013 [6]	Hellström, 1977 [25]	Shaughnessy et al., 2008 [9]	Simmons et al, 1986 [36]	Ximenes et al., 2010 [5]	Lifante-Olivaet al., 2008 [24]	Ximenes et al., 2004 29]	Alonso et al., 2001 [27]	Hurstet al., 1977 [26]	Roberts & Li,1987 [23]
**SAMPLE SELECTION CRITERIA**										
**1) Diagnosis of eating disorders** a) Clinical examination or medical record with validated instrument, or referred from a hospital ★ b)Without clinical examination, or based on self-reports c) No description	★	b	★	★	★	★	★	★	★	★
**2) Representativeness and selection of the patients suffering from eating disorders** a) Patients with eating disorders in a defined catchment area, in a defined hospital or clinic, health maintenance organization, communities or random sample, sample calculation ★ b) Not satisfying requirements in part (a) c) Not stated	★	★	★	★	★	★	★	★	★	★
**COMPARABILITY ON THE BASIS OF THE DESIGN OR ANALYSIS**										
**1) Control for confounders** a) The exposure of interest (tooth erosion) is adjusted for the one confounder ★ b) The exposure of interest (tooth erosion) is adjusted for two or more confounders ★★ c) No description related to the adjustment analysis for confounding factors	★★	★★	c	c	c	c	c	c	c	c
**EVALUATION OF TOOTH EROSION**										
**1) Diagnosis of tooth erosion** a) Clinical examination reporting the use of a tooth erosion index or report of observer agreement (kappa) ★ b) Based on self-reports or not satisfying requirements in part (a) c) No description	★	★	★	★	b	c	b	b	c	c
**2) Response rat**e a) Rate of sample loss ≤20% ★ b) Rate of sample loss >20% c) Not stated	★	★	★	★	★	★	★	★	★	c
**SUMMARY SCORE (Stars)**	6/6 (high)	5/6 (high)	4/6 (high)	4/6 (high)	3/6 (low)	3/6 (low)	3/6 (low)	3/6 (low)	3/6 (low)	2/6 (low)

-. Control (no EDs) vs. anorexia
-. Control (no EDs) vs. bulimia
-. Control (no EDs) vs. bulimia with self-induced vomiting (SIV)
-. Control (no EDs) vs. bulimia without SIV
-. Control (no EDs) vs. any type of EDs
-. EDs without SIV vs. EDs with SIV
-. Control (no ED risk behavior) vs. ED risk behavior

### Methodological Quality Assessment

The quality of the studies was peer-reviewed by APH and PADO using a modified version of the Newcastle-Ottawa Scale for observational studies. Disagreements were resolved by consensus. No cohort study was selected during the analyses as they did not fit the inclusion criteria. No clinical trials were found by electronic and manual search.

A system of points (stars) was given to the eligible categories: sequence generation entries, allocation concealment, blinding, incomplete outcome data, and sample losses. The scale scores varied depending on the study design: for cross-sectional studies it ranged from 0 (lowest grade) to 6 (highest grade) and for case-control studies it ranged from 0 to 10. Studies with scores above the median were classified as high quality studies [12] : >3 for cross-sectional studies and >5 for case-control studies.

Each cross-sectional study could be awarded a maximum of one point for each numbered item, except for the ‘Comparability’ criteria, in which a maximum of two stars could be scored. When referring to the case-control studies, one star could be awarded for each numbered item, except for the items ‘control for confounders’ and ‘diagnosis of tooth erosion’, in which a maximum of two stars could be scored (Tables 3 and 4).

**Table 3:** Case-control studies included in this systematic review presented according to their quality score.

Study	Country (Publication language)	Local setting	Initial sample (final sample)	Gender of the sample	Patients' mean age at dental examination (range in years)	Tooth erosion index (calibration/Kappa)	Type of eating disorder (diagnostic criteria)	Statistics (adjusted for confounders)	Outcomes (OR; 95% CI) or (p-value)	Quality score
Dynesen et al., 2008 [21]	Denmark (English)	Cases: Psychiatric clinic and University Controls: University	40: 20 cases and 20 controls	Female	Cases: 23.8±4 (18–33) Controls: 23.1±2 (20–30)	Larsen et al. modified (K = 0.64)	BN^‡‡^ (DSM IV^‡‡‡‡^)	Multiple regression (age, salivary flow rate and acidic drinks)	TE^††^ score was significantly higher in BN^‡‡^ group compared with the control- group (*p* = 0.019)	9(10)
Ohrn et al., 1999 [35]	Sweden (English)	Cases: Psychiatric clinic Controls: College of nursing	152: 100 cases and 52 controls (133: 81 cases and 52 controls)	Male and female	Cases: 25 (17–47) Controls: 24 (19–41)	Eccles modified by Lussi et al. (NR^†^)	AN^‡^, BN^‡‡^, EDNOS (DSM III-R^‡‡‡^)	*t*-test, Mann-Whitney test and logistic regression (age, number of years suffering from eating disorders)	The eating disorders were associated with TE^††^ severity (<0.001) The period suffering from binge-eating was associated with TE^††^ (*p*<0.01)	9(10)
Järvinen et al., 1991 [28]	Finland (English)	Cases and controls: Metropolitan Helsinki area	206: 106 cases (with TE) and 100 controls (without TE)	Male and female	Cases: 33.1 (13–73) Controls: 36.3 (17–83)	Eccles and Jenkins (1974) (NR^†^)	---	Logistic regression (age, gender)	Prevalence of ED: 7% of the patients from the case-group were suffering from AN^‡^. In the logistic model the practice of vomiting was associated with TE^††^ (OR = 31; 95%CI = 3–300)	9(10)
Johansson et al., 2012 [4]	Sweden (English)	Cases: Eating Disorder Clinic Controls: Public Dental Health Clinic	108: 54 cases and 54 controls	Male and female	Cases:21.5(10–50) Controls: NR^†^	Eccles (1979) modified (training and calibration was performed)	AN^‡^, BN^‡‡^, EDNOS (NR^†^)	Bivariate tests, conditional logistic regression (age)	Eating disorders associated with TE^††^ (OR:8.5; 95%CI: 2.1 – 34.4) Vomiting/binge eating behaviors associated with TE^††^ (OR = 5.5; 95%CI = 1.3–22.9)	8(10)
Emodi-Perlman et al., 2008 [22]	Israel (English)	Cases: Weight and Eating Disorders Center Controls: School of Dental Medicine	136: 86 cases and 50 controls (127: 79 cases and 48 controls)	Female	Cases: 23.46 ± 3.54 (18–35) Controls: 24.58 ±3.01 (18–36)	Johansson et al. (1993) (NR^†^)	AN^‡^, BN^‡‡^, EDNOS (NR^†^)	Chi-square, ANOVA and Tukey's test (no)	Vomiting and non-vomiting groups had higher degree of TE^††^ than controls *(p*<0.001) There was no difference between TE^††^ degree among vomiting and non-vomiting groups (p>0.05)	8(10)
Rytömaa et al., 1998 [20]	Finland (English)	Cases: University Hospital Controls: Universities Dental Services and Colleges	140: 35 cases and 105 controls	Female	Cases: 25.3 ± 6.8 Controls: 25.7 ±7.0	Eccles and Jenkins & Järvinen et al. (K = 0.74–0.94)	BN^‡‡^ (DSM III-R^‡‡‡^)	Chi-square and *t*-test (no)	BN^‡‡^ were associated with TE^††^ (*p*<0.01) Prevalence of TE^††^: 11% among controls; 63% among BN group	8(10)
Robb et al., 1995 [19]	England (English)	Cases: Psychiatric institutions Controls: dental attenders	244: 122 cases and 122 controls	NR^†^	NR^†^	TWI developed by Smith and Knight (NR^†^)	AN^‡^ (purging and restrictive type) and BN^‡‡^ (NR^†^)	Student's *t*-test (no)	AN^‡^ (both types) and BN^‡‡^ were associated with TE^††^ (*p*<0.005)	7(10)
Milosevic & Slade, 1989 [32]	England (English)	Cases and controls: Medical School and School of Dentistry	108: 58 cases and 50 controls	Male and female	Cases:(16–43) Controls: (15–39)	TWI developed by Smith and Knight (NR^†^)	AN^‡^, BN^‡‡^ without SIV^†††^, BN^‡‡^ with SIV^†††^ (DSM III-R^‡‡‡^)	Chi-square, ANOVA (no)	Prevalence of TE^††^: 6% among controls 33% among AN^‡^ patients 28% among BN^‡‡^ without SIV^†††^ patients42% among BN^‡‡^with SIV^†††^ The EDs were associated with TE^††^ (*p*<0.001)	7(10)
Jones & Cleaton-Jones, 1989 [31]	South Africa (English)	Cases and controls: Private dental office	33: 11 cases and 22 controls	Female	Cases:29.8±8.4 Controls: 28.9 ± 9	Own criteria (NR^†^)	BN^‡‡^(NR^†^)	Chi-square (no)	Prevalence of TE^††^: 7% among controls 69% among BN^‡‡^ patients BN^‡‡^ was associated with TE^††^(*p*<0.001)	7(10)
Howat et al., 1990 [18]	USA (English)	Cases: University Eating Disorder Clinic Controls: University (other departments)	20: 10 cases and 10 controls (18: 8 cases and 10 controls)	Female	Cases:24.6 Controls: 22.2	NR^†^	BN^‡‡^ (DSM III-R^‡‡‡^)	*T*-test, Fisher's exact test, ANOVA and Pearson Correlation (no)	No difference between the presence of TE^††^ between the groups (*p*>0.05)	6(10)
Sivolella et al., 2000 [33]	Italy (Italian)	Cases: Eating Disorder Hospital Controls: NR^†^	26: 14 cases and 12 controls	Female	Cases: 23.28 ± 4.9 Controls: 22.58 ± 1.8	NR^†^	AN^‡^, BN^‡‡^ (DSM IV^‡‡‡‡^)	Fisher's exact test (no)	AN^‡^ and BN^‡‡^ were associated with TE^††^ (*p* = 0.009) Prevalence of TE^††^: 41.6% among controls 92.8% among BN^‡‡^ group	5(10)
Greenwood et al., 1988 [30]	Ireland (English)	Cases: University Hospital patients Controls: University Hospital staff	48: 24 cases and 24 controls	Female	AN^‡^ group: (15–24) BN^‡‡^ group: (19–35) Controls: NR^†^	NR^†^	AN^‡^, BN^‡‡^ (NR^†^)	NR^†^	Prevalence of TE^††^: 9% among AN^‡^ patients 30% among BN^‡‡^ patients All patients who SIV^†††^ had TE^††^	5(10)
Touyz et al., 1993 [34]	Australia (English)	NR^†^	45: 30 cases and 15 controls	Female	AN^‡^ group: 20.1 ± 8.3 BN^‡‡^ group: 19.1 ± 3.8 Controls: 22.1 ±3.3	NR^†^	AN^‡^, BN^‡‡^ (DSM III-R^‡‡‡^)	Chi-square and *t*-test (no)	AN^‡^ and BN^‡‡^ were associated with TE^††^ (*p*-value was not reported)	3(10)

AN^‡^  =  anorexia nervosa; BN^‡‡^  =  bulimia nervosa; DSM III- R^‡‡‡^  =  Diagnostic and Statistical Manual of Mental Disorders, 3rd Edition, Revised; DSM IV^‡‡‡‡^  =  Diagnostic and Statistical Manual of Mental Disorders, 4th Edition; NR^†^  =  not reported; TE^††^  =  tooth erosion; SIV^†††^  =  self-induced vomiting.

**Table 4:** Cross-sectional studies included in this systematic review presented according to their quality score.

Study	Country (Publication language)	Local setting	Initial sample (final sample)	Gender of the sample	Patients' mean age at dental examination (range in years)	Tooth erosion index (calibration/Kappa)	Type of eating disorder (diagnostic criteria)	Statistics (adjusted for confounders)	Outcomes (OR; 95% CI) or (p-value)	Quality score
Hermont et al., 2013 [6]	Brazil (English)	Public and private schools	100	Female	(15–18)	O′Sullivan (Kappa = 0.88–0.9)	BITE screening instrument	Chi-square, Fisher's exact test, conditional logistic regression (diet, oral hygiene)	TE^††^ was associated with risk behavior for eating disorder (*p*<0.001) TE^††^ was associated with severe risk behavior for eating disorder(OR = 10.0; 95%CI = 2.5–39.9)	6(6)
Hellström, 1977 [25]	Sweden (English)	Department of Cariology, Karolinska Institute	39	Male and female	Total sample: (14–42) Vomiting group: 26.2±1.2 Non-vomiting group:24.5±1.3	Pindborg (1970) and Eccles & Jenkins(1974) (NR^†^)	AN^‡^ (NR^†^)	Chi-square modified by Fisher (dental caries, gingivitis, salivary factors)	TE^††^ associated with vomiting practices/period suffering from AN^‡^ (*p* = 0.02) Prevalence of TE^††^: 85% among the vomiting group 25% among the non-vomiting group	5(6)
Shaughnessy et al., 2008 [9]	USA (English)	Hospital (Eating Disorder Program)	23	Female	17.6 (14.4–27.2)	Own criteria (NR^†^)	AN^‡^ (DSM IV^‡‡‡‡^)	ANOVA, Student's *t*-test, Pearson correlation (no)	TE^††^ was not detected in any participant	4(6)
Simmons et al, 1986 [36]	USA (English)	University's Eating Disorder Clinic	66	Female	26 (18–34)	Own criteria	BN^‡‡^(DSM III^ψ^)	NR^†^	Prevalence of TE^††^: 37.9% TE^††^ was directly associated with the duration of vomiting practices (p<0.05)	4(6)
Ximenes et al., 2010 [5]	Brazil (English)	Public schools	650	Male and female	(12–16)	NR^†^	EAT-26, BITE screening instruments	Descriptive analysis, chi-square, Student's *t*-test, Levene's *F*-test (no)	TE^††^ was associated with symptoms of eating disorders (*p*<0.001) measured through EAT-26: (OR = 2.52; 95%CI = 1.80–3.52) measured through BITE: (OR = 3.26; 95%CI = 2.35–4.54)	3(6)
Lifante-Oliva et al., 2008 [24]	Spain (English)	Hospital (Eating Disorders Unit)	18 (17)	Female	20.1±5.6 (13–32)	NR^†^	AN^‡^, BN^‡‡^ (DSM IV-R^ψψ^)	NR^†^	Prevalence of TE^††^from vomiting practices: 14.3% among patients with AN^‡^ 70% among patients with BN^‡‡^	3(6)
Ximenes et al., 2004 [29]	Brazil (Portuguese)	Public and private schools	75	Male and female	14 (all sample was 14)	NR^†^	EAT-26 screening instrument	Descriptive analysis (no)	Prevalence of eating disorder risk behavior (cases): 12% Prevalence of TE^††^ in anterior teeth: 100% among cases 28.8% among controls (sample without eating disorder risk behavior)	3(6)
Alonso et al., 2001 [27]	Argentina (Spanish)	Hospital	26	Male and female	15 (12–22)	NR^†^	AN^‡^, BN^‡‡^(DSM IV^‡‡‡‡^)	Fisher's test (no)	TE^††^ was associated with vomiting practices (p = 0.005) Prevalence of TE^††^: 60% among vomiting patients 6% among non-vomiting patients	3(6)
Hurst et al., 1977 [26]	England (English)	Hospital's Psychiatric Department	17	Male and female	Total sample (13–33) Vomiters 24.4 (17–33) Regurgitators 21.4 (13–33) Non-vomiters 21.9 (16–33)	NR^†^	AN^‡^ (NR^†^)	NR^†^	Prevalence of TE^††^: 41% TE^††^ was associated with vomiting and regurgitation (p<0.04)	3(6)
Roberts & Li, 1987 [23]	USA (English)	National Institute of Dental Research	47	Female	AN^‡^: 28 (18–36) BN^‡‡^: 23 (17–34)	NR^†^	AN^‡^, BN^‡‡^ (Feighner, 1972)	NR^†^	Prevalence of TE^††^: 35% among patients with AN^‡^ 33% among patients with BN^‡‡^	2(6)

AN^‡^  =  anorexia nervosa; BN^‡‡^  =  bulimia nervosa; DSM IV^‡‡‡‡^  =  Diagnostic and Statistical Manual of Mental Disorders, 4th Edition; DSM III^ψ^  =  Diagnostic and Statistical Manual of Mental Disorders, 3rd Edition; DSM IV-R^ψψ^  =  Diagnostic and Statistical Manual of Mental Disorders, 4th Edition, Revised; NR^†^  =  not reported; TE^††^  =  tooth erosion; SIV^†††^  =  self-induced vomiting.

### Statistical Methods and Data Synthesis

The Comprehensive Meta-Analysis program, Version 2 [13] was used for meta-analysis. Heterogeneity between studies was evaluated using I^2^ statistics [14]. Meta-analysis was conducted when I^2^ was below 50% and the sensitivity test was conducted when heterogeneity ranged from moderate to high, in order to exclude studies that would increase the heterogeneity. Random effect model was used when heterogeneity was high and fixed effect model for low heterogeneity (0.0%) [14], [15]. Risk measures, 95% confidence interval (CI) and p-value were described in forest plots, and summary risk measures were calculated. Publication bias was not evaluated as there were not enough studies to be grouped in a funnel plot [16], [17].

## Results

### Studies Characteristics

The study selection process is presented in Figure 1. After full text analysis, 23 studies were included in the qualitative synthesis of this systematic review (13 case-control and 10 cross-sectional) and 14 were included in the quantitative synthesis (meta-analysis). A summary of included studies with details including the studies' outcomes as reported and quality appraisal scores is shown in Table 1 and 2.

In general, for case-control studies, cases were recruited from reference centers of EDs and controls were recruited from universities and dental services [4], [18]–[24]. Data for comparison of TE experience among patients suffering from bulimia and anorexia patients was provided by 2 studies [23], [24].

Other studies [25]–[27] compared TE in bulimics with self-induced vomiting and non-vomiting groups. There was only one case-control study [28] that selected patients based on outcome (with TE vs. patients without TE). Three cross-sectional studies [5], [6], [29] evaluated TE in adolescents with ED risk behavior, and the others evaluated adolescents already diagnosed with EDs.

#### Quality assessment

The quality of cross-sectional studies ranged from 2 to 6 (maximum: 6) and 3 to 9 (maximum: 10) for case-control studies (Tables 1, 2, 3 and 4).

### Data Synthesis

#### Comparison of vomiting practices

**Figure 2:**
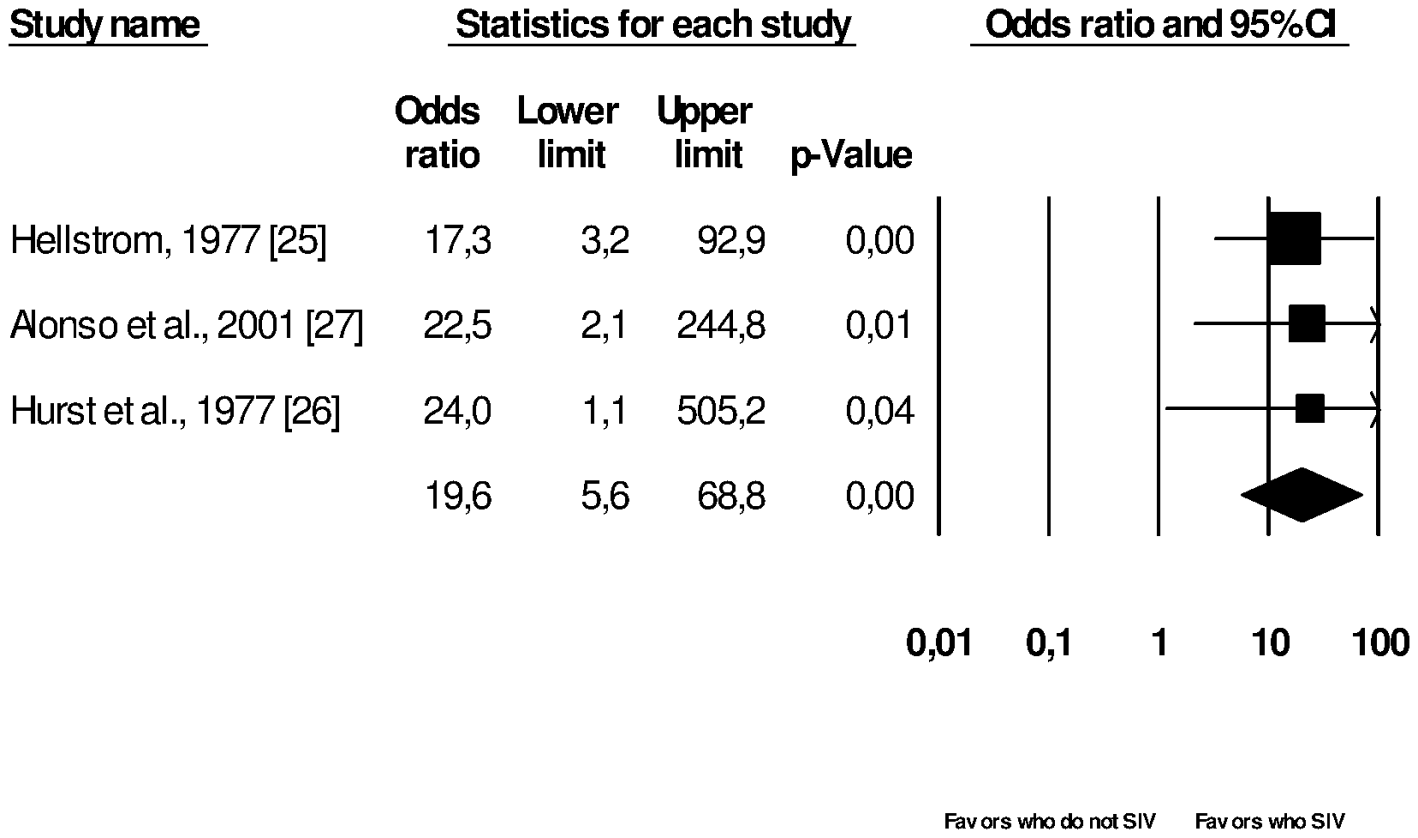
Meta-analysis of three cross-sectional studies associating tooth erosion with patients with eating disorders without self-induced vomiting (SIV) vs. eating disorders with SIV, with statistical significance; I^2^ = 0.0%, fixed effect model used.

Figure 2 shows meta-analysis of three cross-sectional studies [25]–[27] of patients with EDs who SIV vs. patients with EDs that did not SIV and its association with TE. There is a significant association between patients who SIV and increased risk of TE (Summary OR = 19.6, 95%CI = 5.6–68.8).

#### Comparison between types of EDs vs. control (without EDs)

**Figure 3:**
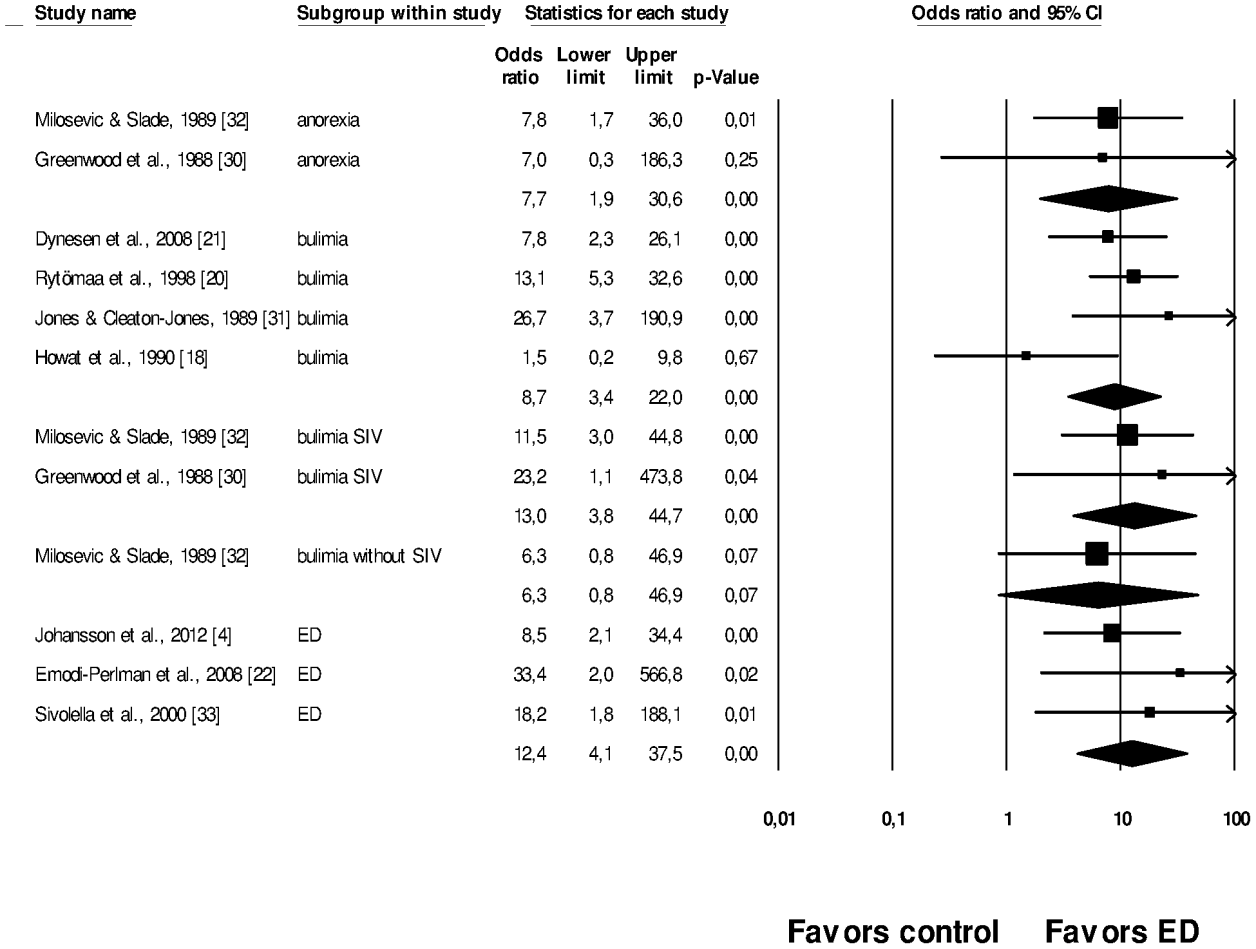
Meta-analysis of nine case-control studies showing twelve different outcomes associating tooth erosion with types of eating disorders (EDs) with or without self-induced vomiting (SIV) vs. controls. Eating disorders were analyzed in subgroups according to each type of ED. Heterogeneity: I^2^ = 0.0% (Anorexia subgroup), I^2^ = 44.0% (Bulimia subgroup), I^2^ = 0.0% (Bulimia with SIV subgroup), I^2^ = 0.0% (Bulimia without SIV subgroup), I^2^ = 0.0% (EDs subgroup), random effect model used.

A total of 9 case-control studies [4], [18], [20]–[22], [30]–[33] were included in this meta-analysis (Figure 3). The analysis was performed in subgroups; twelve outcomes are presented. A sensitivity analysis was performed to decrease heterogeneity. In some studies [4], [22], [33] the types of EDs were not specified, therefore they were grouped in a category called ‘EDs’, according to authors' definitions. The EDs subgroup presented a significant association with TE (Summary OR = 12.4, 95%CI = 4.1–37.5). Also, anorexia (Summary OR = 7.7, 95%CI = 1.9–30.6), bulimia (Summary OR = 8.7, 95%CI = 3.4–22.0), and bulimia with SIV (Summary OR = 13.0, 95%CI = 3.8–44.7) were significantly associated with TE; but not bulimia without SIV (Summary OR = 6.3, 95%CI = 0.8–46.9).

#### ED risk behavior and its association with TE

**Figure 4:**
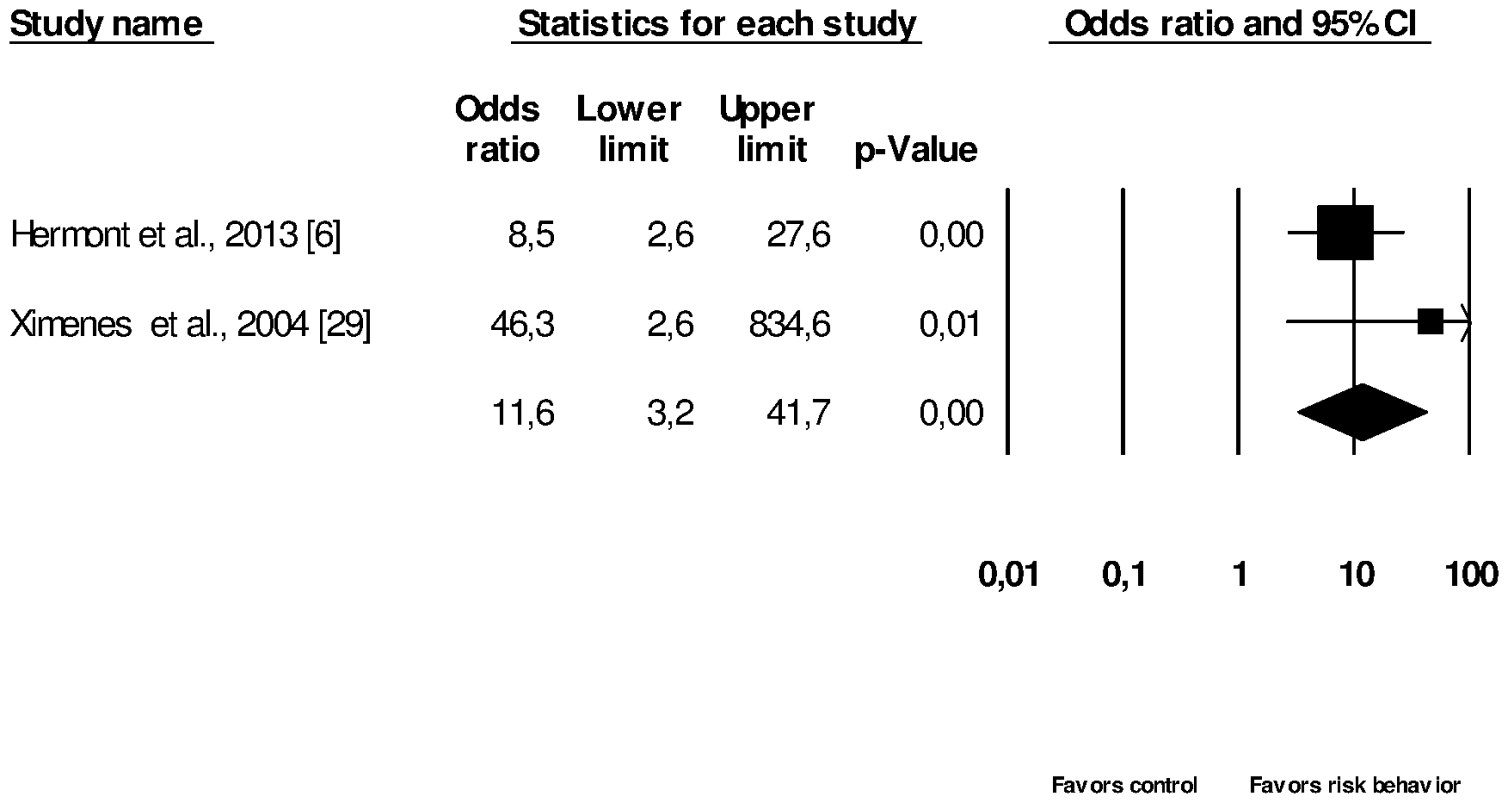
Meta-analysis of two cross-sectional studies associating tooth erosion with eating disorder risk behavior (EDs) vs. control groups (patients without eating disorder risk behavior), with statistical significance; I^2^ = 11.3%, random effect model used.

The meta-analysis and sensitivity analysis of two cross-sectional studies [6], [29] showed a significant association between adolescents with ED risk behavior and TE (Summary OR = 11.6, 95% CI = 3.2–41.7) (Figure 4).

## Discussion

### Assessment of Bias in Included Studies

In the present paper no bias occurred due to language or year of publication, as there was no exclusion related to these reasons. Twenty papers were published in English [4]–[6], [9], [18]–[26], [28], [30]–[32], [34]–[36], and there were also publications in Portuguese [29], Spanish [27] and Italian [33]. The search presented papers published from 1977 [25], [26] to 2013 [6]. A manual search was conducted on reference lists of the studies screened and literature reviews.

### Assessment of Methodological Quality

Among the cross-sectional studies, the major shortcomings involved the data collection process and comparability issues on the basis of the study design or analysis. One study did not mention whether the patients suffering from EDs were diagnosed by clinicians or if validated criteria were used to evaluate the EDs. It also did not mention if the patients were referred from hospitals [25]. Only a few studies reported the criteria used to identify TE or if the examiners had been trained and calibrated [6], [9], [25], [36].

Another shortcoming refers to the external validity of the studies analyzed. During the analysis it was observed that the majority of the studies were conducted among female samples; from the 23 studies included in the meta-analysis, nine included male participants [4], [5], [25]–[29], [32], [35]. Also, the studies had a broad age range, varying from 12 [5] to 83 [28] years.

The absence of controlling for confounders was also observed. Only two studies reported adjusted analysis for confounders, and one controlled the association between TE and EDs for dietary habits and oral hygiene [6]. The other study controlled salivary factors and the duration of oral manifestations and EDs [25]. The studies did not clearly report whether there were sample losses, with only one study including a written description of this aspect [24].

With respect to the case-control studies, one study [28] selected the cases by the presence of TE instead of the manifestation of EDs. Only two studies did not report whether a baseline examination was conducted to ensure that the controls were not suffering from EDs at the beginning of the study [33], [34]. Regarding the oral examinations, four studies did not mention the criteria used for TE diagnosis [18], [30], [33], [34]. Details on the blinding process were also fairly reported as only four studies reported that the examiners were blinded concerning the ED status of the participants during the oral examinations [6], [20], [22], [32].

### Strength of Evidence

The evidence found in the meta-analysis of the case-control studies indicates that patients suffering from different types of EDs, anorexia, bulimia and bulimia with SIV have a greater risk of exhibiting TE in comparison to individuals without such exposure (Figure 3). It was also observed that EDs were significantly associated with the severity of TE (p<0.01) [35]. Only one study did not show this association as TE was not detected in any patient [9]. However, the study pointed out some limitations, such as the small number of participants, which limited the statistical power of the analysis performed [9]. Bulimia and anorexia were significantly associated with TE (Figure 3). However, the authors did not specify whether the disorders involved vomiting practices. The association of bulimia, anorexia and TE was also highlighted by other studies [19], [34].

There was a statistical association of bulimia with SIV and TE, but not for bulimia without SIV (Figure 3). This reinforces the hypothesis that purging techniques are crucial cofactors for the occurrence of TE, which results from a chronically acidic oral environment [4]. This can be reinforced by Figure 2, which shows that patients suffering from EDs who SIV had a significant greater risk of TE when compared to patients who did not SIV. In this case, EDs were not specified by the authors. Such an association was also observed in other studies [28], [36].

Nevertheless, analysis from Figures 2 and 3 involved a small number of studies. For example, in Figure 3, the subgroup of bulimia presented four studies and all other subgroups were composed of fewer studies. Also, Figure 2 included only three studies. The small number of studies analyzed can decrease the statistical power of the tests. In this research it can be observed that some results (Figures 2, 3 and 4) present large confidence intervals, decreasing the statistical power and the precision of the estimated population effect size [37]. One possible explanation for the low statistical power resulting from such intervals may rely on the small sample of some studies.

Furthermore, the methodological quality can influence the meta-analysis interpretation [12]. The studies included in Figures 2 and 3 were very heterogeneous. Figure 3 included studies with quality ranging from 5 to 9 points; Figure 2 included two studies that scored 3 points [26], [27] and one that scored 5 points [25] (Tables 2 and 4). It is well known that the type of quality assessment scale used affects the analysis and the conclusions of meta-analytic studies, therefore the use of other quality scales could have resulted in different outcomes [12].

Several studies [4], [19]–[21], [31]–[35] which were conducted in different settings and using different methods showed similar findings related to the possible causal relationship between EDs and TE. Specificity, temporality, and dose-response relationships were also observed. Some studies observed that the years of exposure to EDs and vomiting practices influenced the risk of TE [35], [36]. Nevertheless, only cross-sectional and case-control studies were included in this systematic review. No cohort study was found. This highlights the need of prospective cohort studies, which could provide the highest strength of association to confirm this scientific evidence.

The association between the ED risk behavior and the presence of TE was investigated by three studies [5], [6], [29]. One of the studies [5] did not enter the meta-analysis, as it was excluded during the sensitivity analysis. When analyzing the forest plot it can be observed that although the studies' meta-analysis showed a significant association between these variables, the confidence intervals were large, decreasing the statistical power [37]. Moreover, the studies' quality assessment revealed a high degree of heterogeneity; one study scored 3 points [29] and the other scored 6 points [6] (Tables 2 and 4). The methodological heterogeneity evaluated by the quality assessment scale can influence the meta-analysis interpretation [12] and the strength of evidence.

Inferential causal conclusions associating the ED risk behavior and TE cannot be drawn due to the studies' cross-sectional design and the limited number of studies which were evaluated in the meta-analysis (Figure 4).

The consistency related to the possible causal relationship between ED risk behavior and TE is weak. There are only 3 studies analyzing this issue and two of them were conducted in the same place [5], [29]. Furthermore, by analyzing the literature it is still not possible to draw any conclusion related to the specificity, temporality, dose-response, experimental evidence, biological plausibility, coherence or analogy between both conditions considering only three cross-sectional studies. Up to the present time, there is still a lack of solid evidence for an increased TE risk due to EDs, once there is no low risk of bias reporting a large effect.

In the present systematic review it was not possible to evaluate the publication bias as there were not enough studies to be grouped in a funnel plot [17]. However, there seems to be a tendency to report a positive association between EDs and TE.

## Conclusions

Purging practices seem to increase the risk of TE. Nevertheless, there is a significant lack of scientific evidence to fulfill the basic criteria of causation between both conditions. Moreover, the present systematic review does reveal that to date there is no solid evidence in support of the postulated causal role of EDs in the occurrence of TE.

It is important to conduct prospective cohort studies in this area in order to investigate such evidence. Special attention should be given to studies on ED risk behavior, as the main goal should be to detect subclinical cases and avoid the onset of EDs and further comorbidities.

## Supporting Information

Table S1 **List of titles selected for full text analysis and the reasons for exclusion.**
(DOC)

Checklist S1 **PRISMA 2009 Checklist.**
(DOC)
