# Supplementary material for: Tooth Erosion and Eating Disorders: A Systematic Review and Meta-Analysis
Source: PLoS One. 2014 Nov 7;9(11):e111123. doi: 10.1371/journal.pone.0111123 (PMC4224381; doi:10.1371/journal.pone.0111123)
Supplement: Table S1 — List of titles selected for full text analysis and the reasons for exclusion. (DOC) [file pone.0111123.s001.doc]

| **Reference** | **Classification** |
| --- | --- |
| 1. Vreven J, Vermeersch G, Mainguet P. Dental lesions associated with exogenous and endogenous acids. Acta Endoscopica 2008; 38(3):263-281. | NOT SELECTED: Review and has no association with tooth erosion |
| 1. Spigset O. Oral symptoms in bulimia nervosa. A survey of 34 cases. Acta Odontol Scand 1991; 49(6):335-339. | NOT SELECTED: Review and epidemiological study, nevertheless it has no relevant association between tooth erosion and eating disorders |
| 1. Lussi A, Hellwig E, Zero D, Jaeggi T. Erosive tooth wear: diagnosis, risk factors and prevention. Am J Dent 2006; 19(6):319-325. | NOT SELECTED  Review (about tooth wear) |
| 1. Buczkowska-Radlinska J, Kaczmarek W, Tyszler L, Mikolajczyk E, Fraczak B. [Dental status in patients with eating disorders]. Ann Acad Med Stetin 2007; 53(1):90-93. | NOT SELECTED  Review (concerning the oral status and dental complications in patients with eating disorders) |
| 1. Schroeder PL, Filler SJ, Ramirez B, Lazarchik DA, Vaezi MF, Richter JE. Dental Erosion and Acid Reflux Disease. Annals of Internal Medicine 1995; 122(11):809-815. | NOT SELECTED  Cross-sectional study concerning tooth erosion and gastroesophageal disease |
| 1. Robb ND, Cruwys E, Smith BG. Regurgitation erosion as a possible cause of tooth wear in ancient British populations. Arch Oral Biol 1991; 36(8):595-602. | NOT SELECTED  The study reports no association between tooth erosion and eating disorders |
| 1. Sivasithamparam K, Young WG, Jirattanasopa V, Priest J, Khan F, Harbrow D et al. Dental erosion in asthma: a case-control study from south east Queensland. Aust Dent J 2002; 47(4):298-303. | NOT SELECTED  Case-control study concerning tooth erosion and asthma/ GERD |
| 1. Valena V, Young WG. Dental erosion patterns from intrinsic acid regurgitation and vomiting. Aust Dent J 2002; 47(2):106-115. | NOT SELECTED  The study reports no association between tooth erosion and eating disorders |
| 1. Frydrych AM, Davies GR, McDermott BM. Eating disorders and oral health: a review of the literature. Aust Dent J 2005; 50(1):6-15. | NOT SELECTED  Review concerning dental implications and eating disorders) |
| 1. Taji S, Seow WK. A literature review of dental erosion in children. Aust Dent J 2010; 55(4):358-367. | NOT SELECTED  Review concerning tooth erosion among children) |
| 1. Linnett V, Seow WK, Connor F, Shepherd R. Oral health of children with gastro-esophageal reflux disease: A controlled study. Australian Dental Journal 2002; 47(2):156-162. | NOT SELECTED  Case-control study concerning dental health among children with GERD |
| 1. Wang P, Lin HC, Chen JH, Liang HY. The prevalence of dental erosion and associated risk factors in 12-13-year-old school children in Southern China. BMC Public Health 2010; 10:478. | NOT SELECTED  Cross-sectional study concerning tooth erosion among children and related risk factors |
| 1. Smith BG, Knight JK. Dental erosion due to anorexia nervosa with bulimia. Br Dent J 1982; 152(6):187. | NOT SELECTED  Letter to the editor |
| 1. Andrews FF. Dental erosion due to anorexia nervosa with bulimia. Br Dent J 1982; 152(3):89-90. | NOT SELECTED  Case-report concerning tooth erosion and eating disorders |
| 1. Smith BG, Knight JK. A comparison of patterns of tooth wear with aetiological factors. Br Dent J 1984; 157(1):16-19. | NOT SELECTED  Cross-sectional study concerning tooth wear but reports no relevant association with eating disorders |
| 1. Pothier PE. Dental erosion in anorectic patients. Br Dent J 1995; 178(12):445. | NOT SELECTED  Letter to the Editor |
| 1. Shaw L, Smith AJ. Dental erosion--the problem and some practical solutions. Br Dent J 1999; 186(3):115-118. | NOT SELECTED  Review  (Tooth erosion prevalence, risk factors and related issues) |
| 1. Milosevic A, Bardsley PF, Taylor S. Epidemiological studies of tooth wear and dental erosion in 14-year old children in North West England. Part 2: The association of diet and habits. Br Dent J 2004; 197(8):479-483. | NOT SELECTED  Cross-sectional study concerning tooth erosion among schoolchildren |
| 1. Dougall A, Fiske J. Access to special care dentistry, part 6. Special care dentistry services for young people. Br Dent J 2008; 205(5):235-249. | NOT SELECTED  Review |
| 1. Serra MC, Messias DC, Turssi CP. Control of erosive tooth wear: possibilities and rationale. Braz Oral Res 2009; 23 Suppl 1:49-55. | NOT SELECTED  Review  (Tooth erosion prevalence, risk factors and related issues) |
| 1. Bartlett DW, Evans DF, Anggiansah A, Smith BGN. A study of the association between gastro-oesophageal reflux and palatal dental erosion. British Dental Journal 1996; 181(4):125-131. | NOT SELECTED  Cross-sectional study concerning tooth erosion and gastro-oesophageal reflux |
| 1. Kelleher M, Bishop K. Tooth surface loss: an overview. British Dental Journal 1999; 186(2):61-66. | NOT SELECTED  Review  (Tooth surface loss) |
| 1. Milosevic A, Dawson LJ. Salivary factors in vomiting bulimics with and without pathological tooth wear. Caries Res 1996; 30(5):361-366. | NOT SELECTED  There are no association between eating disorders and tooth erosion. Nevertheless, there are associations with Ph, salivary issues |
| 1. Ganss C, Schlechtriemen M, Klimek J. Dental erosions in subjects living on a raw food diet. Caries Res 1999; 33(1):74-80. | NOT SELECTED  Cross-sectional study concerning tooth erosion and diet (raw food) |
| 1. Lussi A, Schaffner M. Progression of and risk factors for dental erosion and wedge-shaped defects over a 6-year period. Caries Res 2000; 34(2):182-187. | NOT SELECTED  Cross-sectional study concerning tooth erosion and wedge-shaped defects, does not mention eating disorders |
| 1. Mathew T, Casamassimo PS, Hayes JR. Relationship between sports drinks and dental erosion in 304 university athletes in Columbus, Ohio, USA. Caries Res 2002; 36(4):281-287. | NOT SELECTED  Cross-sectional study concerning tooth erosion and sports drinks, does not mention eating disorders |
| 1. van Rijkom HM, Truin GJ, Frencken JE, Konig KG, 't Hof MA, Bronkhorst EM et al. Prevalence, distribution and background variables of smooth-bordered tooth wear in teenagers in the hague, the Netherlands. Caries Res 2002; 36(2):147-154. | NOT SELECTED  Cross-sectional study concerning tooth wear among teenagers |
| 1. Lussi A, Jaeggi T, Zero D. The role of diet in the aetiology of dental erosion. Caries Res 2004; 38 Suppl 1:34-44. | NOT SELECTED  Review  (Tooth erosion and diet) |
| 1. Bardolia P, Burnside G, Ashcroft A, Milosevic A, Goodfellow SA, Rolfe EA et al. Prevalence and risk indicators of erosion in thirteen- to fourteen-year-olds on the Isle of Man. Caries Res 2010; 44(2):165-168. | NOT SELECTED  Cross-sectional study concerning the prevalence of tooth erosion in a determined sample and dietary risk factors |
| 1. Ratnayake N, Ekanayake L. Risk indicators for tooth wear in Sri Lankan adolescents. Caries Res 2010; 44(1):14-19. | NOT SELECTED  Cross-sectional study: reports no association between tooth erosion and eating disorders |
| 1. Jarvinen V, Rytomaa I, Meurman JH. Location of Dental Erosion in A Referred Population. Caries Research 1992; 26(5):391-396. | NOT SELECTED  Cross-sectional study: reports no association between tooth erosion and eating disorders |
| 1. Jensdottir T, Arnadottir IB, Thorsdottir I, Bardow A, Gudmundsson K, Theodors A et al. Relationship between dental erosion, soft drink consumption, and gastroesophageal reflux among Icelanders. Clin Oral Investig 2004; 8(2):91-96. | NOT SELECTED  Cross-sectional study: reports no association between tooth erosion and eating disorders |
| 1. Lussi A, Jaeggi T. Erosion--diagnosis and risk factors. Investig 2008; 12 Suppl 1:S5-13. | NOT SELECTED  Review  Reports no association between tooth erosion and eating disorders |
| 1. Rosenthal P, Rosenthal R. Tooth enamel erosion from vomiting treated with an acrylic sealant. Clin Pediatr (Phila) 1983; 22(12):818. | NOT SELECTED  The study is about dental materials and there are no association between tooth erosion and eating disorders |
| 1. Milosevic A, Lennon MA, Fear SC. Risk factors associated with tooth wear in teenagers: a case control study. Community Dent Health 1997; 14(3):143-147. | NOT SELECTED  Case-control study - no association between tooth erosion and eating disorders |
| 1. El Karim IA, Sanhouri NM, Hashim NT, Ziada HM. Dental erosion among 12-14 year old school children in Khartoum: a pilot study. Community Dent Health 2007; 24(3):176-180. | NOT SELECTED  Cross-sectional study: reports no association between tooth erosion and eating disorders |
| 1. Sanhouri NM, Ziada HM, Ahmed GI, Kamis AH. Tooth surface loss, prevalence and associated risk factors among 12-14 years school children in Khartoum State, Sudan. Community Dent Health 2010; 27(4):206-212. | NOT SELECTED  Cross-sectional study: reports no association between tooth erosion and eating disorders |
| 1. Jiang H, Du MQ, Huang W, Peng B, Bian Z, Tai BJ. The prevalence of and risk factors for non-carious cervical lesions in adults in Hubei Province, China. Community Dent Health 2011; 28(1):22-28. | NOT SELECTED  Cross-sectional study: reports no association between tooth erosion and eating disorders |
| 1. Lussi A, Schaffner M, Hotz P, Suter P. Dental erosion in a population of Swiss adults. Community Dent Oral Epidemiol 1991; 19(5):286-290. | NOT SELECTED  Cross-sectional study: reports no association between tooth erosion and eating disorders |
| 1. Bader JD, McClure F, Scurria MS, Shugars DA, Heymann HO. Case-control study of non-carious cervical lesions. Community Dent Oral Epidemiol 1996; 24(4):286-291. | NOT SELECTED  Case-control study - no association between tooth erosion and eating disorders |
| 1. Al Majed I, Maguire A, Murray JJ. Risk factors for dental erosion in 5-6 year old and 12-14 year old boys in Saudi Arabia. Community Dent Oral Epidemiol 2002; 30(1):38-46. | NOT SELECTED  Cross-sectional study: reports no association between tooth erosion and eating disorders |
| 1. Al Malik MI, Holt RD, Bedi R. Erosion, caries and rampant caries in preschool children in Jeddah, Saudi Arabia. Community Dent Oral Epidemiol 2002; 30(1):16-23. | NOT SELECTED  Cross-sectional study: reports no association between tooth erosion and eating disorders |
| 1. Woodmansey KF. Managing erosion associated with bulimia nervosa. Dent Today 1996; 15(10):86-89. | NOT SELECTED  Review  Reports no association between tooth erosion and eating disorders.  The article re -examines a possible treatment/ management of patients with tooth erosion |
| 1. Shaw L, Smith A. Erosion in children: an increasing clinical problem? Dent Update 1994; 21(3):103-106. | NOT SELECTED  Review  Reports no association between tooth erosion and eating disorders.  The article examines tooth erosion issues and suggests ways of overcoming it |
| 1. Ashcroft A, Milosevic A. The eating disorders: 1. Current scientific understanding and dental implications. Dent Update 2007; 34(9):544-50, 553. | NOT SELECTED  Review  Concerning some dental implications of eating disorders and related issues |
| 1. Ashcroft A, Milosevic A. The eating disorders: 2. Behavioural and dental management. Dent Update 2007; 34(10):612-620. | NOT SELECTED  Review  Concerning the dental management of eating disorders patients |
| 1. Montecchi PP, Custureri V, Polimeni A, Cordaro M, Costa L, Marinucci S et al. Oral manifestations in a group of young patients with anorexia nervosa. Eat Weight Disord 2003; 8(2):164-167. | NOT SELECTED  Epidemiological study concerning oral manifestations in patients with binge-eating/ purging anorexia, but it presents only descriptive data |
| 1. Nunn JH. Prevalence of dental erosion and the implications for oral health. Eur J Oral Sci 1996; 104(2 ( Pt 2)):156-161. | NOT SELECTED  Review  Reports no association between tooth erosion and eating disorders. |
| 1. ten Cate JM, Imfeld T. Dental erosion, summary. Eur J Oral Sci 1996; 104(2 ( Pt 2)):241-244. | NOT SELECTED  Review  Concerning the tooth erosion issues |
| 1. Scheutzel P. Etiology of dental erosion--intrinsic factors. Eur J Oral Sci 1996; 104(2 ( Pt 2)):178-190. | NOT SELECTED  Review  Concerning the aetiology of tooth erosion (intrinsic factors) |
| 1. O'Sullivan EA, Curzon ME, Roberts GJ, Milla PJ, Stringer MD. Gastroesophageal reflux in children and its relationship to erosion of primary and permanent teeth. Eur J Oral Sci 1998; 106(3):765-769. | NOT SELECTED  Cross-sectional study: reports no association between tooth erosion and eating disorders |
| 1. Ohrn R, Angmar-Mansson B. Oral status of 35 subjects with eating disorders--a 1-year study. Eur J Oral Sci 2000; 108(4):275-280. | NOT SELECTED  Longitudinal study concerning dental status among eating disorder patients, but it was not possible to collect data appropriately |
| 1. Willumsen T, Graugaard PK. Dental fear, regularity of dental attendance and subjective evaluation of dental erosion in women with eating disorders. Eur J Oral Sci 2005; 113(4):297-302. | NOT SELECTED  Cross-sectional study: evaluates tooth erosion among patients with eating disorders but tooth erosion was detected by means of self-report |
| 1. da Silva AM, Oakley DA, Hemmings KW, Newman HN, Watkins S. Psychosocial factors and tooth wear with a significant component of attrition. Eur J Prosthodont Restor Dent 1997; 5(2):51-55. | NOT SELECTED  Case-control study: reports no association between tooth erosion and eating disorders. |
| 1. Burnett CA, Hussey DL, Clifford TJ. Presentation, diagnosis and initial management of patients referred to a hospital tooth wear clinic. Eur J Prosthodont Restor Dent 2001; 9(1):5-7. | NOT SELECTED  Cross-sectional study: study characterized a group of patients with oral complains. Only 4 were diagnosed as eating disorder and the study made no statistical association with dental erosion. |
| 1. Harwood P, Newton T. Dental Aspects of Bulimia-Nervosa - Implications for the Health-Care Team. European Eating Disorders Review 1995; 3(2):93-102. | NOT SELECTED  Review  Concerning the dental aspects of bulimia nervosa |
| 1. Zero DT. Etiology of dental erosion - Extrinsic factors. European Journal of Oral Sciences 1996; 104(2):162-177. | NOT SELECTED  Review  Concerning the extrinsic causes of tooth erosion |
| 1. Imfeld T. Dental erosion. Definition, classification and links. European Journal of Oral Sciences 1996; 104(2):151-155. | NOT SELECTED  Review  About tooth wear (definition classification and related issues) |
| 1. Harrison JL, George LA, Cheatham JL, Zinn J. Dental effects and management of bulimia nervosa. Gen Dent 1985; 33(1):65-68. | NOT SELECTED  Review and case reports  Concerning the management and dental implications among bulimic patients |
| 1. Woodmansey KF. Recognition of bulimia nervosa in dental patients: implications for dental care providers. Gen Dent 2000; 48(1):48-52. | NOT SELECTED  Review  Concerning bulimia nervosa and oral signs |
| 1. Shipley S, Taylor K, Mitchell W. Identifying causes of dental erosion. Gen Dent 2005; 53(1):73-75. | NOT SELECTED  Review  Concerning tooth erosion patterns and patients management |
| 1. Bassiouny MA. Clinical features and differential diagnosis of erosion lesions: systemic etiologies. Gen Dent 2010; 58(3):244-255. | NOT SELECTED  Review  Concerning tooth erosion and its systemic etiologies |
| 1. Williams D, Croucher R, Marcenes W, O'Farrell M. The prevalence of dental erosion in the maxillary incisors of 14-year-old schoolchildren living in Tower Hamlets and Hackney, London, UK. Int Dent J 1999; 49(4):211-216. | SELECTED  Cross-sectional study: evaluates the prevalence of tooth erosion among a selected sample |
| 1. Ayers KM, Drummond BK, Thomson WM, Kieser JA. Risk indicators for tooth wear in New Zealand school children. Int Dent J 2002; 52(1):41-46. | NOT SELECTED  Cross-sectional study: reports no association between tooth erosion and eating disorders |
| 1. Oginni O, Olusile AO. The prevalence, aetiology and clinical appearance of tooth wear: the Nigerian experience. Int Dent J 2002; 52(4):268-272. | NOT SELECTED  Cross-sectional study: reports no association between tooth erosion and eating disorders |
| 1. Bartlett DW. The role of erosion in tooth wear: aetiology, prevention and management. Int Dent J 2005; 55(4 Suppl 1):277-284. | NOT SELECTED  Review  Concerning tooth wear (erosion) issues |
| 1. Young WG. Tooth wear: diet analysis and advice. Int Dent J 2005; 55(2):68-72. | NOT SELECTED  Review  Concerning tooth wear (erosion) issues and preventive strategies |
| 1. Milosevic A, Brodie DA, Slade PD. Dental erosion, oral hygiene, and nutrition in eating disorders. Int J Eat Disord 1997; 21(2):195-199. | NOT SELECTED  It was not possible to extract data about tooth erosion related to eating disorders. The specific findings concerning this issue are not clear. |
| 1. Shaw L, O'Sullivan E. UK National Clinical Guidelines in Paediatric Dentistry. Diagnosis and prevention of dental erosion in children. Int J Paediatr Dent 2000; 10(4):356-365. | NOT SELECTED  Review  Concerning tooth erosion among children |
| 1. Gurgel CV, Rios D, de Oliveira TM, Tessarolli V, Carvalho FP, Machado MA. Risk factors for dental erosion in a group of 12- and 16-year-old Brazilian schoolchildren. Int J Paediatr Dent 2011; 21(1):50-57. | NOT SELECTED  Cross-sectional study: reports no association between tooth erosion and eating disorders.  Reports tooth erosion issues among schoolchildren in Brazil |
| 1. Bernhardt O, Gesch D, Splieth C, Schwahn C, Mack F, Kocher T et al. Risk factors for high occlusal wear scores in a population-based sample: results of the Study of Health in Pomerania (SHIP). Int J Prosthodont 2004; 17(3):333-339. | NOT SELECTED  Cross-sectional study: reports risk factors for tooth wear |
| 1. Philipp E, Willershausenzonnchen B, Hamm G, Pirke KM. Oral and Dental Characteristics in Bulimic and Anorectic Patients. International Journal of Eating Disorders 1991; 10(4):423-431. | NOT SELECTED  Case-control study concerning oral status among eating disorders patients, but it was not possible to collect data appropriately |
| 1. Hattab FN, Yassin OM. Etiology and diagnosis of tooth wear: A literature review and presentation of selected cases. International Journal of Prosthodontics 2000; 13(2):101-107. | NOT SELECTED  Review and case reports  Concerning the etiology and diagnosis of tooth wear |
| 1. Abrams RA, Ruff JC. Oral signs and symptoms in the diagnosis of bulimia. J Am Dent Assoc 1986; 113(5):761-764. | NOT SELECTED  Review  Concerning the oral implications of bulimia nervosa |
| 1. Burkhart N, Roberts M, Alexander M, Dodds A. Communicating effectively with patients suspected of having bulimia nervosa. J Am Dent Assoc 2005; 136(8):1130-1137. | NOT SELECTED  Review  Concerning the assessment of tooth erosion among bulimics |
| 1. Chadwick RG, Mitchell HL, Manton SL, Ward S, Ogston S, Brown R. Maxillary incisor palatal erosion: no correlation with dietary variables? J Clin Pediatr Dent 2005; 29(2):157-163. | NOT SELECTED  Cross-sectional study: reports no association between tooth erosion and eating disorders |
| 1. Ahmed H, Durr-E-Sadaf, Rahman M. Factors associated with Non-Carious Cervical Lesions (NCCLs) in teeth. J Coll Physicians Surg Pak 2009; 19(5):279-282. | NOT SELECTED  Cross-sectional study: reports no association between tooth erosion and eating disorders |
| 1. Gandara BK, Truelove EL. Diagnosis and management of dental erosion. J Contemp Dent Pract 1999; 1(1):16-23. | NOT SELECTED  Review  Concerning tooth erosion issues. No correlation to eating disorders is mentioned |
| 1. Aranha AC, Eduardo CP, Cordas TA. Eating disorders. Part I: Psychiatric diagnosis and dental implications. J Contemp Dent Pract 2008; 9(6):73-81. | NOT SELECTED  Review  Concerning eating disorders and oral implications |
| 1. El Aidi H, Bronkhorst EM, Huysmans MC, Truin GJ. Dynamics of tooth erosion in adolescents: a 3-year longitudinal study. J Dent 2010; 38(2):131-137. | NOT SELECTED  Longitudinal study  Concerning tooth erosion among adolescents and related issues |
| 1. Ritter AV. Eating disorders and oral health. J Esthet Restor Dent 2006; 18(2):114. | NOT SELECTED  Review |
| 1. Bretz W. Dental fear, dental attendance, and self-report of dental erosion appear to be altered in women with eating disorders. J Evid Based Dent Pract 2006; 6(3):246. | NOT SELECTED  It’s a letter to the editor |
| 1. Toro M, Zunt S, Fontana M. Dental erosion: a multi-factorial condition. J Indiana Dent Assoc 2006; 85(2):6-13. | NOT SELECTED  Review and case reports  Concerning tooth erosion issues |
| 1. Cataldo E, Santis HR. A clinico-pathologic presentation. Tooth erosion. J Mass Dent Soc 1991; 40(2):53, 86. | NOT SELECTED  Case-report |
| 1. Mitchell JE. Dental complications of bulimia nervosa. J N J Dent Assoc 1991; 62(1):73-75. | NOT SELECTED  Review  Concerning the medical and dental consequences of bulimia nervosa |
| 1. ODA patient's page. Bulimia and your teeth. J Okla Dent Assoc 2009; 100(7):7. | NOT SELECTED  Review |
| 1. Chuajedong P, Kedjarune-Leggat U, Kertpon V, Chongsuvivatwong V, Benjakul P. Associated factors of tooth wear in southern Thailand. J Oral Rehabil 2002; 29(10):997-1002. | NOT SELECTED  Cross-sectional study: reports the risk factors for tooth wear |
| 1. Dahshan A, Patel H, Delaney J, Wuerth A, Thomas R, Tolia V. Gastroesophageal reflux disease and dental erosion in children. J Pediatr 2002; 140(4):474-478. | NOT SELECTED  Cross-sectional study: reports no association between tooth erosion and eating disorders.  The article reports tooth erosion among children with GERD |
| 1. Smith BG, Bartlett DW, Robb ND. The prevalence, etiology and management of tooth wear in the United Kingdom. J Prosthet Dent 1997; 78(4):367-372. | NOT SELECTED  Review  Concerning the prevalence of tooth wear in the UK |
| 1. Mangueira DF, Sampaio FC, Oliveira AF. Association between socioeconomic factors and dental erosion in Brazilian schoolchildren. J Public Health Dent 2009; 69(4):254-259. | NOT SELECTED  Cross-sectional study: reports no association between tooth erosion and eating disorders.  The article reports the potential risk factors for tooth erosion among Brazilian schoolchildren |
| 1. Owens BM, Gallien GS, Schuman NJ, Turner JE. Perimylolysis of the permanent dentition in an adolescent. J Tenn Dent Assoc 1997; 77(1):26-29. | NOT SELECTED  Case reports  Concerning tooth erosion among adolescents |
| 1. Drago CJ. Dental diagnosis and treatment of chronic vomiting patients. J Wis Dent Assoc 1985; 61(4):291-293. | NOT SELECTED  Case reports  Concerning dental implications and management of chronic vomiting patients |
| 1. Aine L, Baer M, Maki M. Dental Erosions Caused by Gastroesophageal Reflux Disease in Children. Journal of Dentistry for Children 1993; 60(3):210-214. | NOT SELECTED  Cross-sectional study: reports no association between tooth erosion and eating disorders.  The article reports dental implications among children who suffer from GERD |
| 1. Tylenda CA, Roberts MW, Elin RJ, Li SH, Altemus M. Bulimia-Nervosa - Its Effect on Salivary Chemistry. Journal of the American Dental Association 1991; 122(7):37-41. | NOT SELECTED  Case –control study  Concerning eating disorders and implications in the salivary chemistry |
| 1. Seremet M. Clinical research of dental erosion. Med Arh 1995; 49(1-2):35-38. | NOT SELECTED  Review |
| 1. Bartlett D. Intrinsic causes of erosion. Monogr Oral Sci 2006; 20:119-139. | NOT SELECTED  Review  Concerning intrinsic causes of tooth erosion |
| 1. Lussi A, Jaeggi T. Dental erosion in children. Monogr Oral Sci 2006; 20:140-151. | NOT SELECTED  Review  Concerning tooth erosion among children |
| 1. Lussi A, Hellwig E. Risk assessment and preventive measures. Monogr Oral Sci 2006; 20:190-199. | NOT SELECTED  Review  Concerning erosive tooth wear and associated factors |
| 1. Mahoney EK, Kilpatrick NM. Dental erosion: part 1. Aetiology and prevalence of dental erosion. N Z Dent J 2003; 99(2):33-41. | NOT SELECTED  Review  Concerning the aetiology and prevalence of tooth erosion |
| 1. Spigset O. [Bulimia and destruction of dental hard tissues]. Nor Tannlaegeforen Tid 1987; 97(12):508-511. | NOT SELECTED  Review |
| 1. Chan DC, Browning WD, Pohjola R, Hackman S, Myers ML. Predictors of non-carious loss of cervical tooth tissues. Oper Dent 2006; 31(1):84-88. | NOT SELECTED  Cross-sectional study: concerning non-carious cervical loss and risky behaviors |
| 1. Bohmer CJ, Klinkenberg-Knol EC, Niezen-de Boer MC, Meuwissen PR, Meuwissen SG. Dental erosions and gastro-oesophageal reflux disease in institutionalized intellectually disabled individuals. Oral Dis 1997; 3(4):272-275. | NOT SELECTED  Cross-sectional study: concerning tooth erosion and reflux among intellectually disabled individuals |
| 1. Wiegand A, Muller J, Werner C, Attin T. Prevalence of erosive tooth wear and associated risk factors in 2-7-year-old German kindergarten children. Oral Dis 2006; 12(2):117-124. | NOT SELECTED  Cross-sectional study: concerning erosive tooth wear in kindergarten children |
| 1. Lo Russo L, Campisi G, Di Fede O, Di Liberto C, Panzarella V, Lo Muzio L. Oral manifestations of eating disorders: a critical review. Oral Diseases 2008; 14(6):479-484. | NOT SELECTED  Review  Concerning oral manifestations of eating disorders |
| 1. Silva MAGS, Damante JH, Stipp ACM, Tolentino MM, Carlotto PR, Fleury RN. Gastroesophageal reflux disease: New oral findings. Oral Surgery Oral Medicine Oral Pathology Oral Radiology and Endodontics 2001; 91(3):301-310. | NOT SELECTED  Cross-sectional study: investigates the effects of GERD on dentition |
| 1. Deery C, Wagner ML, Longbottom C, Simon R, Nugent ZJ. The prevalence of dental erosion in a United States and a United Kingdom sample of adolescents. Pediatr Dent 2000; 22(6):505-510. | NOT SELECTED  Cross-sectional study: concerning the prevalence of tooth erosion |
| 1. Roberts MW, Tylenda CA. Dental aspects of anorexia and bulimia nervosa. Pediatrician 1989; 16(3-4):178-184. | NOT SELECTED  Review  Concerning dental aspects of eating disorders patients |
| 1. Bouquot JE, Seime RJ. Bulimia nervosa: dental perspectives. Pract Periodontics Aesthet Dent 1997; 9(6):655-663. | NOT SELECTED  Review  Concerning dental implications of bulimia nervosa |
| 1. Raigrodski AJ, Dogan S. Concepts and considerations of tooth wear: part I--the chemical component. Pract Proced Aesthet Dent 2007; 19(8):511-512. | NOT SELECTED  Review  Concerning tooth wear issues |
| 1. Ediger M. Do the eating habits of anorexics and bulimics have an effect on their oral health? Probe 1994; 28(4):139-140. | NOT SELECTED  Review |
| 1. Paszynska E, Limanowska-Shaw H, Slopien A, Rajewski A. [Evaluation of oral health in bulimia nervosa]. Psychiatr Pol 2006; 40(1):109-118. | NOT SELECTED  Case-control study concerning dental health and bulimia nervosa, but it was not possible to collect data appropriately |
| 1. Schluter N, Ganss C, Klimek J, Zeeck A. Dental erosion in eating disorders - Diagnosis, prevention and therapy. Psychotherapeut 2006; 51(6):465-473. | NOT SELECTED  Review  Concerning tooth erosion and eating disorders |
| 1. Litonjua LA, Andreana S, Bush PJ, Cohen RE. Tooth wear: Attrition, erosion, and abrasion. Quintessence International 2003; 34(6):435-446. | NOT SELECTED  Review  Concerning tooth wear issues/ types/ patterns |
| 1. Willershausen B, Joseph W, Zimmermann C. [Characteristic oral changes in patients with anorexia nervosa and bulimia nervosa]. Quintessenz 1990. 41(9):1513-1518. | NOT SELECTED  Case-control study concerning eating disorders and tooth erosion, but it was not possible to collect data once it refers to the tooth surface and it was not possible to group data by subjects |
| 1. Curca M, Danila I. [Clinical study on the distribution of tooth wear of the adult population]. Rev Med Chir Soc Med Nat Iasi 2010; 114(3):870-873. | NOT SELECTED  Cross-sectional study: concerning the prevalence of tooth wear |
| 1. Lussi A, Schaffner M, Hotz P, Suter P. [The erosion of dental hard substance. Its epidemiology, clinical appearance, risk factors and preventive rules]. Schweiz Monatsschr Zahnmed 1992; 102(3):320-329. | NOT SELECTED  Review |
| 1. Stich H. [Erosion. Clinical aspects--diagnosis--risk factors--prevention--therapy]. Schweiz Monatsschr Zahnmed 2005; 115(10):917-946. | NOT SELECTED  Review  Concerning clinical appearance and the risk factors for the development of erosive lesions |
| 1. Johansson AK, Johansson A, Stan V, Ohlson CG. Silicone sealers, acetic acid vapours and dental erosion: a work-related risk? Swed Dent J 2005; 29(2):61-69. | NOT SELECTED  Case –control study  Concerning risk factors for tooth erosion related with the workplace |
| 1. Johansson AK. On dental erosion and associated factors. Swed Dent J Suppl 2002;(156):1-77. | NOT SELECTED  Cross-sectional study: concerning the prevalence of tooth erosion an d related issues |
| 1. Harper-Mallonee L, Wright JM, Allen CM. Oral and maxillofacial pathology case of the month. Erosion secondary to bulimia nervosa. Tex Dent J 2006; 123(10):976, 980-976, 981. | NOT SELECTED  Case-report |
| 1. Mungia R, Zarzabal LA, Dang SC, Baez M, Stookey GK, Brown JP. Epidemiologic survey of erosive tooth wear in San Antonio, Texas. Tex Dent J 2009; 126(11):1097-1109. | NOT SELECTED  Cross-sectional study: concerning the prevalence of tooth wear |
| 1. Waszkiel D. [Diet as an important factor in the etiology of dental erosions]. Wiad Lek 2004; 57(11-12):647-652. | NOT SELECTED  Cross-sectional study: concerning dental erosion and dietary issues |
| 1. Zachariasen RD. Oral manifestations of bulimia nervosa. Women Health 1995; 22(4):67-76. | NOT SELECTED  Review  Concerning oral manifestations of bulimia nervosa |
| 1. Willershausen B, Philipp E, Pirke KM, Fichter M. [Oral complications in patients with anorexia nervosa and bulimia nervosa]. Zahn Mund Kieferheilkd Zentralbl 1990; 78(4):293-299. | NOT SELECTED  Case-control study concerning eating disorders and tooth erosion, but it was not possible to collect data once it refers to the tooth surface and it was not possible to group the data by subjects |
| 1. Kavitha PR, Vivek P, Hegde AM. Eating disorders and their implications on oral health--role of dentists. J Clin Pediatr Dent 2011;36(2):155-60. | NOT SELECTED  Review (oral manifestations of eating disorders) |
| 1. Li H, Zou Y, Ding G. Dietary factors associated with dental erosion: a meta-analysis. PLoS One 2012;7(8):e42626. | NOT SELECTED  Review (diet-related factors for dental erosion) |
| 1. Marder MZ. Oral medicine and the teenage patient. Dent Today 2011 Jul;30(7):90-3. | NOT SELECTED  Review (oral manifestations of eating disorders) |
| 1. Mulic A, Skudutyte-Rysstad R, Tveit AB, Skaare AB. Risk indicators for dental erosive wear among 18-yr-old subjects in Oslo, Norway. Eur J Oral Sci 2012 Dec;120(6):531-8. | NOT SELECTED  The article reports the association between vomiting and dental erosive wear, but the study does not specify in the methods section the etiology of the vomiting habits (it is unclear whether there is an association between tooth erosion with eating disorders). |
| 1. Resch M, Nagy A. [Stomatologic complications of eating disorders]. Orv Hetil 2012 Nov 11;153(45):1779-86. | NOT SELECTED  Review (oral manifestations of eating disorders) |
| | 1. Araújo, Juliana Julianelli de. | | --- | | Avaliação da prevalência de desgaste dentario em pacientes portadores de transtornos alimentares / Evaluation of prevalence of tooth wear in patients with ating disorders. | | Bauru; s.n; 2007. xl,205 p. ilus, tab. | | NOT SELECTED  Epidemiological study concerning the oral changes in eating disorders patients, but the type of tooth wear was not specified (it is not clear the association between eating disorders with the erosive tooth wear) |
| 1. [Manifestaciones dentales en pacientes con anorexia y bulimia tipo compulsivo purgativo/ Dental manifestations of patients with anorexia and compulsive purgative type bulimia](http://pesquisa.bvsalud.org/regional/resources/lil-565644)   Thomas, Yeily; Ibáñez, Édgar; Serrano, Claudia; Teherán, Dannys  [CES Odontol](http://portal.revistas.bvs.br/transf.php?xsl=xsl/titles.xsl&xml=http://catserver.bireme.br/cgi-bin/wxis1660.exe/?IsisScript=../cgi-bin/catrevistas/catrevistas.xis|database_name=TITLES|list_type=title|cat_name=ALL|from=1|count=50&lang=pt&comefrom=home&home=false&task=show_magazines&request_made_adv_search=false&lang=pt&show_adv_search=false&help_file=/help_pt.htm&connector=ET&search_exp=CES Odontol); 21(2): 33-38, jul.-dic. 2008. ilus, tab. | NOT SELECTED  Epidemiological study concerning the tooth erosion and eating disorders, but it presents only descriptive data |
| 1. [Trastornos de la conducta alimentaria en adolescentes: componente salud bucal de un encuadre interdisciplinario/ Eating behavior disorders in adolescents: oral health component in an interdisciplinary approach](http://pesquisa.bvsalud.org/regional/resources/lil-351960)   García de Valente, María Shirley; Bordoni, Noemí Emma; Rozensztejn, Ruth; Armatta, Ana  [Bol AAON](http://portal.revistas.bvs.br/transf.php?xsl=xsl/titles.xsl&xml=http://catserver.bireme.br/cgi-bin/wxis1660.exe/?IsisScript=../cgi-bin/catrevistas/catrevistas.xis|database_name=TITLES|list_type=title|cat_name=ALL|from=1|count=50&lang=pt&comefrom=home&home=false&task=show_magazines&request_made_adv_search=false&lang=pt&show_adv_search=false&help_file=/help_pt.htm&connector=ET&search_exp=Bol AAON); 32(3): 23-27, sept.-dic. 2003. tab, graf | NOT SELECTED  Epidemiological study concerning the tooth erosion and eating disorders, but it presents only descriptive data |
| 1. [Manifestaciones dentarias y en restauraciones de pacientes bulímicos y anoréxicos purgativos/ Dental and restorative manifestations in bulimic and anorexic patients](http://pesquisa.bvsalud.org/regional/resources/lil-500115)   Zeman, Liliana.  [Rev Asoc Odontol Argent](http://portal.revistas.bvs.br/transf.php?xsl=xsl/titles.xsl&xml=http://catserver.bireme.br/cgi-bin/wxis1660.exe/?IsisScript=../cgi-bin/catrevistas/catrevistas.xis|database_name=TITLES|list_type=title|cat_name=ALL|from=1|count=50&lang=pt&comefrom=home&home=false&task=show_magazines&request_made_adv_search=false&lang=pt&show_adv_search=false&help_file=/help_pt.htm&connector=ET&search_exp=Rev Asoc Odontol Argent); 96(2): 153-157, abr.-mayo 2008. tab, graf | NOT SELECTED  Epidemiological study concerning to tooth erosion and eating disorders, but it presents only descriptive data |
| 1. Hamasha AA, Zawaideh FI, Al-Hadithy RT. Risk indicators associated with dental erosion among Jordanian school children aged 12-14 years of age. Int J Paediatr Dent 2013 Feb 24. | NOT SELECTED  Unrelated epidemiological study (the article reports the association between vomiting episodes and tooth erosion, but the study does not specify in the methods section the etiology of the vomiting habits (it is unclear whether there is an association between tooth erosion with eating disorders). |
| 1. Wentz E, Gillberg IC, Anckarsater H, Gillberg C, Rastam M. Somatic problems and self-injurious behaviour 18 years after teenage-onset anorexia nervosa. Eur Child Adolesc Psychiatry 2012 Aug;21(8):421-32. | NOT SELECTED  Case-control study concerning somatic problems of anorexia nervosa, but tooth erosion was detected by means of self-report |
| 1. Zachariasen RD. Oral signs and symptoms of bulimia nervosa. J Gt Houst Dent Soc 1997; 69(1):31-34. | NOT SELECTED  Review (concerning oral manifestations of bulimia nervosa) |
